# miR-10a is aberrantly overexpressed in *Nucleophosmin1 *mutated acute myeloid leukaemia and its suppression induces cell death

**DOI:** 10.1186/1476-4598-11-8

**Published:** 2012-02-20

**Authors:** Adam Bryant, Catalina A Palma, Vivek Jayaswal, Yee Wa Yang, Mark Lutherborrow, David DF Ma

**Affiliations:** 1 Blood, Stem Cells and Cancer Research. St Vincent's Centre for Applied Medical Research, St Vincent's Hospital, Sydney, NSW, Australia; 2 School of Mathematics and Statistics, Sydney Bioinformatics Centre for Mathematical Biology, University of Sydney, Sydney, NSW, Australia; 3 St Vincent's Clinical School, Faculty of Medicine, University of New South Wales, Sydney, NSW, Australia

**Keywords:** Acute myeloid leukaemia (AML), Nucleophosphmin1, microRNA, miR-10a, microarray, Cell death

## Abstract

**Background:**

Acute myeloid leukaemia (AML) with nucleophosmin-1 (*NPM1*) mutation is a major subtype of AML. The *NPM1 *mutation induces a myeloproliferative disorder, but evidence indicates that other insults are necessary for the development of AML. We utilised microRNA microarrays and functional assays to determine if microRNA dysregulation could be involved in the pathogenesis of in *NPM1 *mutated (*NPM1^mut^*)-AML.

**Results:**

We used a stringent locked nucleic acid (LNA) based microRNA microarray platform to profile bone marrow samples of patients with normal karyotype AML. A panel of five microRNAs dichotomised AML patients according to their *NPM1 *mutational status. miR-10a, let-7b and let-7c were significantly over-expressed, while miR-130a and miR-335 were under-expressed in *NPM1^mut^*-AML when compared to *NPM1^wildtype^*-AML. Of these, miR-10a is the most differentially expressed in *NPM1^mut^*-AML versus *NPM1^wildtype^*-AML (> 10 fold higher as confirmed by qRT-PCR). To investigate the functions of miR-10a, the OCI-AML3 cell line was utilised, which is the only commercially available cell line bearing *NPM1^mut^*. OCI-AML3 cells were firstly demonstrated to have a similarly high miR-10a expression to primary *NPM1^mut^*-AML patient samples. Inhibition of miR-10a expression by miRCURY LNA Inhibitors (Exiqon) in these cells resulted in increased cell death as assessed by MTS, cell cycle and Annexin-V assays and reduced clonogenic capacity, indicative of an involvement in leukaemic cell survival. *In silico *filtering of bioinformatically predicted targets of miR-10a identified a number of potential mRNA targets with annotated functions in haematopoiesis, cell growth and apoptosis. Lucferase reporter assays confirmed a number of these putative tumorogenic genes that are miR-10a suppressible including *KLF4 *and *RB1CC1*. This provides a potential mechanism for the pathogenic role of miR-10a in *NPM1^mut^*-AML.

**Conclusions:**

This study provides, for the first time, *in vitro *evidence of a pro-survival role of miR-10a in *NPM1^mut^*-AML, that it may contribute to the pathogenesis of *NPM1^mut^*-AML and identifies putative tumorogenic targets.

## Background

Acute myeloid leukaemia (AML) represents the convergent outcome a number of genetic abnormalities that have consequence in crucial cellular pathways of haematopoiesis. With an increasing knowledge of cellular processes and an accompanying improvement in our ability to interrogate these pathways, a spectrum of recurrent genetic abnormalities relevant to AML has become increasingly apparent. While most AML cases have at least one detectable genetic mutation potentially responsible for their pathogenesis, there remains a significant minority in which no abnormality is detectable [1].

MicroRNA-mediated post-transcriptional control of gene expression is a relatively newly discovered mechanism of cellular regulation that could account for some of the gaps in our knowledge of AML pathogenesis [2]. Through their repressive action on complementary sites in 3' untranslated regions (3'UTR) of target genes [2], these short 19-25 nucleotide RNA species are important in numerous processes including haematopoietic stem cell maintenance [3] and progenitor self-renewal [4], myeloid differentiation [5-7], cell cycle and proliferation [8,9], apoptosis [10,11] and gene methylation [12]. All of these pathways are of potential relevance to AML pathogenesis if dysregulated.

*NPM1*-mutated *AML (NPM1^mut^-AML) *accounts for approximately 30% of cases of adult AML (and up to 60% of AML with normal karyotype) [13]. Recently *NPM1^mut ^*activation has been found to initiate a myeloproliferative disorder after knock-in into mouse haematopoietic stem cells, however co-expression with a secondary mutation was proposed to be needed for overt AML development [14]. Global microRNA expression was assayed in a cohort of normal karyotype AML (NK-AML) using a stringent LNA-based microarray platform. We showed that among other differentially expressed microRNAs miR-10a, but not miR-10b, was significantly markedly over-expressed in *NPM1^mut^*-AML versus AML not bearing *NPM1 *gene insertions (*NPM1^WT^*-AML). It is also demonstrated that the only available *NPM1 *mutated cell line, OCI-AML3, exhibits high miR-10a expression. We have demonstrated that knockdown of over-expressed miR-10a in these cells resulted in reduced cellular survival and clonogenic growth. Using luciferase reporter analysis, we confirmed several miR-10a suppressible target genes located in key cellular pathways of significance to AML. Together, these findings suggest miR-10a may provide a pro-survival signal contributing to the pathogenesis of *NPM1*^mut^-AML.

## Results

### microRNA profiling of AML samples by *NPM1 *mutational status

**Figure 1:**
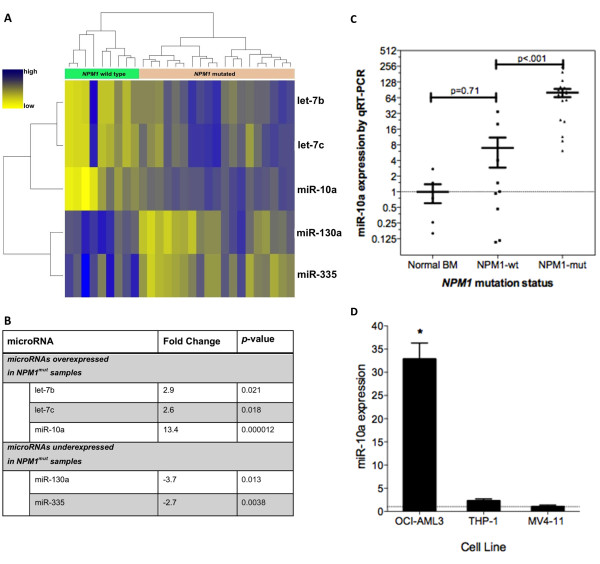
**Unique microRNA signature of *NPM1^mut ^*AML is characterised by miR-10a over-expression**. **A **Hierarchical cluster analysis of 28 AML samples according to *NPM1 *mutational status, with over-expression of miR-10a, let-7b, let-7c and under-expression of miR-130a and miR-335, with accompanying fold change **B**. **C **Validation of miR-10a expression by qRT-PCR in normal bone marrow, *NPM1^wt^-*AML and *NPM1^mut^*-AML respectively, with values presented normalised to the mean of the normal bone marrow samples. **D **miR-10a expression by qRT-PCR in selected malignant cell lines: MV4-11, OCI-AML3 (myelomonoblastic) and THP-1 (monoblastic). The expression values are depicted relative to that of MV4-11 cells, which had the lowest miR-10a expression. Error bars denote SEM. *p < 0.05 by Unpaired Two-tailed T-test.

Analysis of microRNA expression of NK-AML samples demonstrated a clear pattern of clustered expression according to *NPM1 *mutational status, with three microRNAs over-expressed in *NPM1^mut^*-AML (miR-10a, let-7b and let-7c) and two microRNAs under-expressed (miR-130a and miR-335) (Figure 1A and 1B).

Furthermore, miR-10a was the most over-expressed microRNA with a FC of 19.6 as compared to normal bone marrow (Additional file 1: Table S1). miR-10a was 13.4 fold over-expressed in *NPM1^mut^*-AML compared to *NPM1^WT^*-AML samples, indicative of the potential importance of miR-10a in *NPM1^mut^*-AML (Figure 1B). In comparison for miR-10b (whose sequence only differs from miR-10a by a single base pair), the magnitude of the FC was only 1.4. Further to this, the expression of ratio of miR-10a to miR-10b in the *NPM1^mut ^*samples was 53:1. This indicates that miR-10a and not miR-10b is the dominantly expressed gene in *NPM1^mut^*-AML.

qRT-PCR was used to confirm that miR-10a expression is significantly higher in the *NPM1^mut^*-AML group compared to both *NPM1^WT^*-AML and normal BM samples (mean FC values of 79.9, 7.0 and 1.0 respectively) (Figure 1C). Thus while there was some overlap of miR-10a expression between *NPM1^mut ^*and *NPM1^WT^*-AML samples, high levels of miR-10a expression were very specific for *NPM1^mut ^*-AML. miR-10a expression was not significantly DE between normal bone marrow and *NPM1^WT^-AML*.

### Inhibition of miR-10a expression by antisense LNA oligonucleotides

miR-10a was expressed at higher levels in OCI-AML3 cells than other myelomonocytic leukeamic cell lines (Figure 1D). These results indicate that OCI-AML3 cells recapitulate the miR-10a expression of their parental primary leukaemic sample and may therefore serve as a useful model for study of miR-10a over-expression and its relationship with the *NPM1 *mutation.

**Figure 2:**
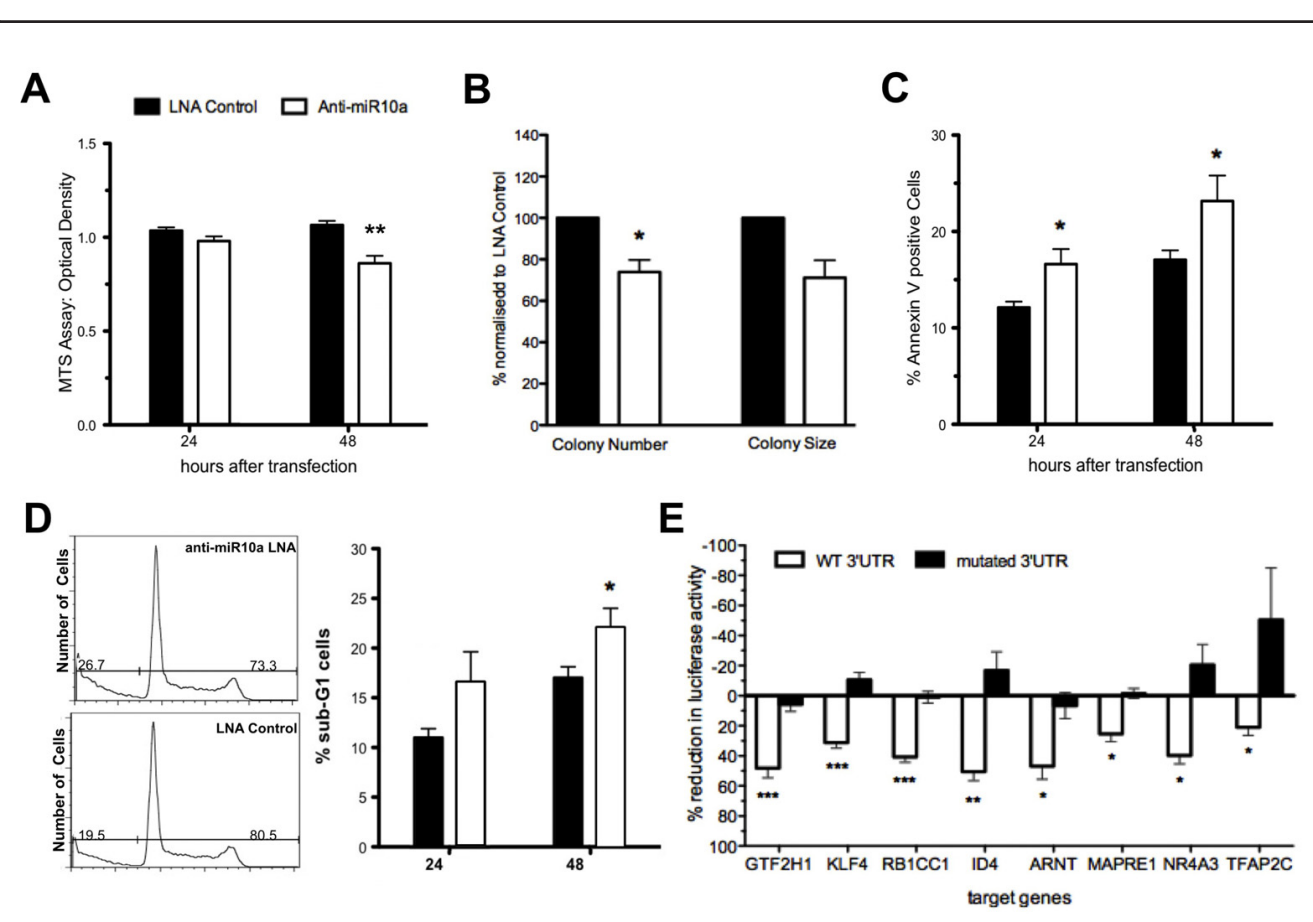
**Functional effects of miR-10a**. **A**. Transfection of OCI-AML3 cells with anti-miR10a LNA resulted in 19% reduction in cell count at 48 h as determined by MTS assay. **B **Assessment of clonogenic potential found a significant decrease in mean colony number (left), and a trend towards reduced Colony Size (right). Values are normalised to Scrambled LNA Control. **C **A significant increase in the number of Annexin-V positive events at 24 and 48 h. **D **Cell cycle analysis demonstrated an increase in the proportion of cells in the SubG1 fraction at 24 and 48 h post miR-10a knockdown. Black bars = Scrambled LNA Control, White bars = Anti-miR10a LNA transfected OCI-AML3 cells. **E **Confirmation of predicted miR-10a gene targets by luciferase reporter assay: *ARNT, GTFH1, ID4, KLF4, MAPRE1, NR4A3, RB1CC1 *and *TFAP2C *were confirmed to be miR-10a repressible by this assay. White bars = miR-10a repression of 3'UTR of the gene of interest, Black bars = miR10a repression of mutated miR-10a binding site on the 3'UTR of the gene of interest. All values were subject to normalisation to renilla luciferase activity of a non-specific transfection control. Error bars denote SEM. For all experiments n ≥ 3, statistical significance determined by Two-Tailed T-Test. *p < 0.05, **p < 0.01, *** *p *< 0.001.

Transfection of OCI-AML3 cells with anti-miR-10a LNA resulted in knockdown of miR-10a expression as assessed by qRT-PCR and by reporter assay (Additional file 2: Figure S1). As determined by MTS assay, inhibition of miR-10a resulted in significant reduction in cell count (19%, *p *< 0.01) at 48 h compared to treatment with a non-targeting LNA (Figure 2A).

miR-10a knockdown resulted in increased proportions of Annexin-V positive events compared to control treated cells by 34.9% and 39.3% at 24 and 48 h respectively, indicating increased cell death (Figure 2C, n = 4). Furthermore, knockdown of miR-10a resulted in a 29.4% increase in the number of cells in the sub-G_1 _fraction of the cell cycle (Figure 2D, n = 4) also indicating an increase in cell death. Caspase-3 activation was not detected in the assays analysed in response to miR-10a knockdown (data not shown), indicating that the increased cell death was not due to classical activation of apoptosis. Further flow cytometry analysis did not show a perturbation in cell cycle composition or cellular proliferation (PI and BrdU respectively, data not shown). miR-10a knockdown was not found to have an influence on the monocytic differentiation potential of OCI-AML3 cells, with no significant difference in CD14, CD15 or CD11b expression after 48 or 96 h of induction culture with 1,25-dihydroxyvitaminD3 (Additional file 3: Figure S2).

Clonogenic potential of OCI-AML3 cells (Figure 2B) demonstrated that knock-down in miR-10a levels resulted in a reduced clonogenic capacity, with significant reduction in colony number after 14 days (26.1%, *p *< 0.05), with an associated trend towards smaller colony size. These *in vitro *studies suggest miR10a has a pro-survival role in the *NPM1^mut^*-AML cell line.

### Prediction and functional analysis of miR-10a targets

To identify genes potentially responsible for these functional findings, we utilised the miRanda, PicTar and TargetScan algorithms to predict mRNA targets of miR-10a. This search yielded 976, 133 and 143 mRNA targets respectively for a total 1071 unique targets. 44 genes were simultaneously predicted by all three algorythms (Table 1), and these will be referred to as stringently predicted targets.

**Table 1:** miR-10a targets predicted by each of miRanda, PicTar and TargetScan.

miR-10a gene targets predicted by miRanda, TargetScan and PicTar			
ACTG1	DOCK11	MAPRE1	SDC1

ANKRD12	DVL3	NARG1	SFRS1

ARNT	ELAVL2	NCOA6	SLC25A1

ASXL1	FHL3	NCOR2	SLC38A2

BCL6	FXR2	NR4A3	SMAP1

BCL2L2	GRM3	NR5A2	SON

BTBD11	GTF2H1	PAFAH1B1	SVOP

BTBD14B	HNRPK	POPDC2	TFAP2C

CNNM4	HOXD10	PURB	USF2

CTDSPL	ID4	RAP2A	WNK3

DAZAP1	IL1RAPL1	RB1CC1	ZMYND11

Of a total of unique 1071 mRNA targets predicted across the three algorithms, 44 were predicted to be targeted by miR-10a by all three

A list of genes that are known to be or could be hypothesised to be dysregulated in AML was compiled, and interrogated back to the permissively identified set of potential miR-10a targets. Several resources were utilised to compile this catalogue (as indicated in Table 2). Advantage was also taken of published *NPM1^mut^*-AML gene expression profiling data [15-17] by compiling a list of genes down-regulated in *NPM1^mut^*-AML on the microarrays. Correlating these genes with the 1071 predicted miR-10a targets revealed potential miR-10a gene targets in each group as listed in Table 2. Of particular note, the Sanger Cancer Census genes *ARNT, BCL6 *and *NR4A3*, as well at the G.O.C. myeloid-related gene *NCOA6 *(GO:0003099), each were also stringently predicted miR-10a targets of the 3 predictive algorithms. With respect to the genes down-regulated in *NPM1^mut^*-AML, *CENTD1, ITM2A, RARA *and *SPARC *each possess permissively identified miR-10a binding sites.

**Table 2:** miR-10a targets in pathways of potential relevance to AML.

Cancer Census Genes		KEGG AML	KEGG apoptosis	KEGG cell cycle	G.O.C. -myeloid related	*NPM1^mu^*^t^-AML gene array
***n = 366***		***n = 57***	***n = 62***	***n = 124***	***n = 80***	***n = 53***

**ARNT**	MLLT6	PIK3CA	BAX	CCNB3	KLF4	CENTD1

**BCL6**	NF2	RARA	BCL2L11	CDK4	**NCOA6**	ITM2A

CDK4	NONO		CASP9	CDKN2A	PURB	RARA

CREBBP	**NR4A3**		FOXO3	CREBBP	RASGRP4	SPARC

ERBB2	PDE4DIP		IRAK4	ORC1L		

ERCC3	PIK3CA			ORC2L		

FANCD2	PTEN			ORDC5L		

FOXO3	RARA					

FUS	RECQL4					

HMGA2	SSX2					

MLLT10	WRN					

The genes listed in this table are those in each pathway that are also one of the 1071 permissively identified miR-10a targets. *n *refers to the number of genes in each pathway interrogated for miR-10a targets. The genes linked to each of the heading in this table are listed in supplemental section. The final column refers to genes that were down regulated in three mRNA gene expression profiling studies of *NPM1^mut^*-AML [15-17]. The genes denoted in bold are those that were also stringently identified by all 3 prediction programs. KEGG- Kyoto Encyclopaedia of Genes and Genomes. GOC.- Gene Ontology Consortium

Twelve genes were selected from the genes listed in Tables 1 and 2 for further analysis by reporter assay: *ARNT, CTDSPL, ID4, GTF2H1, KLF4, MAPRE1, NFIX, NR4A3, NCOA6, NCOR2, RB1CC1, TFAP2C*. Each of these genes has previously been demonstrated to be involved in malignant processes through their down-regulation. Reporter assay confirmed *ARNT, GTFH1, ID4, KLF4, MAPRE1, NR4A3, RB1CC1 *and *TFAP2c *to be miR-10a repressible genes, with further evidence of targetting specificity provided by site directed mutagenesis of the miR-10a binding site (Figure 2E).

## Discussion

miR-10 family members are now implicated in the malignant transformation across a range of tissues through altered expression including breast cancer [18-21], colon and oesophageal cancer [22,23], glioblastoma [24-26], hepatocellular carcinoma [27], melanoma [18,28], neurofibromatosis [29], pancreatic cancer [30] and urothelial carcinoma [31]. In murine models of breast carcinoma miR-10b has been demonstrated to have a crucial role in metastasis [19] with the pro-metastatic effect repressible by systemic antisense inhibition of miR-10b [21]. A similar role has also been demonstrated in oesophageal cell lines [22]. miR-10a may have a role in initial oncogenic transformation events evidenced by the finding that miR-10a enhances the transformation of NIH-3T3 cells by RAS-V12 [32]. Contrary to these findings miR-10a is actually down-regulated in chronic myeloid leukaemia (CML) CD34+ cells and over-expression of miR-10a retards the growth of KU812 cells (CML cell line) [33].

miR-10a first appeared in reference to AML when it was individually selected along with miR-10b for expression analysis in a cohort of AML cases [34], by virtue of its residence within HOX clusters known to be overexpressed in AML [35]. In a subsequent study, miR-10a and miR-10b were found to be overexpressed (4.7 and 3.1 fold respectively) in NK-AML versus non NK-AML [36,37]. Thus, miR-10 family expression appeared to be reflective of the HOX over-expression characteristic of NK-AML. The HOX expression profile itself appears more specific to *NPM1^mut^*-AML which has the gene expression reminiscent of the haematopoietic stem cell [15].

Garzon et al., (2008) also compared microRNA expression between *NPM1^mut^*-AML versus *NPM1^WT^*-AML. They found that a microRNA signature, dominated by miR-10a, miR-10b and miR-100 over-expression in *NPM1^mut^*-AML, was able to accurately discriminate these AML subtypes. miR-10 family over-expression was also noted in two subsequent studies undertaking a similar comparison [38,39], but each utilising the alternative platform of a multiplexed TaqMan^® ^MicroRNA Assay [40].

In each of the earlier publications miR-10a and miR-10b were reported be co-overexpressed with a similar FC. However, it may be difficult to confidently discriminate miR-10a from miR-10b expression, since they differ at only a single nucleotide in the centre of the mature microRNA outside the seed site. While miR-10a and miR-10b share the same active 7 nucleotide seed site and therefore a vastly overlapping target profile, each is transcribed from distinct genomic locations and are likely to be controlled by their own cis-regulatory networks. The current study utilised a stringent LNA-based array which has a capacity to resolve miR-10a from miR-10b expression with cross-hybridisation of less than 10% for each probe. We found that while miR-10a was overexpressed in *NPM1^mut^-AML *to a degree concordant with the earlier studies (13.4 fold), miR-10b was much more modestly DE (1.4 fold). Further to this, the expression ratio of miR-10a to miR-10b in the *NPM1*-mutated samples was 54:1. This suggests that miR-10a is the dominantly expressed family member in *NPM1-*AML cases, which verifies Garzon et al., finding [41] and is confirmed by a recent study by Ovchrenko et al. [42].

The normal physiological role of miR-10a is uncertain. miR-10a is deeply conserved both with respect to its sequence as well as its location within the HOXB cluster [43,44]. The finding that several HOX genes including HOXA1 [45], HOXA3 [46], HOXD4 [47] and HOXD10 [19] are repressible targets of miR-10 suggests that miR-10 and HOX genes form an interconnected regulatory network with an important role in embryonic development. To date, minimal evidence exists to indicate that miR-10 has a physiological role in normal haematopoiesis, substantiated only by the observation that miR-10a is down-regulated as haematopoietic precursors mature towards certain differentiated progeny, such as megakaryocytes [48] or lymphocytes [49].

To further evaluate the potential role of miR-10a over-expression in *NPM1^mut^*-AML, functional studies are required to address the possibility the over-expression may represent a passenger phenomenon consequent on a permissive chromatin configuration encompassing the HOXB locus. This is particularly important, since *NPM1^mut^*-AML is known to possess a HOX up-regulated expression signature, although it is noted that miR-10a is not overexpressed in *MLL-*deregulated AMLs [38] which similarly upregulate HOX genes [50,51].

We conducted functional studies in the *NPM1^mut ^*OCI-AML3 cell line. We have demonstrated in this study that knockdown of miR-10a overexpression in these cells lead to a decreased cell count after 48 hours in culture. Increased Annexin-V positive events but no change in cell cycling or proliferation was noted at 24 and 48 h. However, Caspase-3 was not activated, which suggests that the reduced cell survival occurs by a mechanism other than classic apoptosis, such as necrosis, or via activation of Apoptosis-Inducible Factor leading to Caspase 3- independent apoptosis [52]. Interestingly, assessment of the clonogenic potential of OCI-AML3 cells by growth on semi-solid media found a decrease in the clonogenic potential of cells with miR-10a knockdown, suggesting a possible role for miR-10a in self renewal pathways.

We acknowledge that this study is constrained by the current lack of *NPM1^mut^*-AML cell lines, and future studies should determine the functional role of miR-10a by manipulating its expression levels in primary cells from AML patients, both with *NPM1^wt ^*and *NPM1^mut^*, which has its own technical challenges. The interaction between miR-10a and *NPM1^mut ^*needs to be defined. Evaluation of the leukaemogenic qualities of miR-10a, and its connection with the *NPM^mut^*, in murine models might address the unanswered issues raised by this study.

Prior to this study, only a short list of miR-10a repressible targets had been experimentally confirmed including *Pbp1, RAN *[32], *USF2 *[33] and the previously mentioned HOX genes. We wished to identify miR-10a gene targets which may explain the in vitro functional findings induced by suppression of miR-10a. Particular attention was paid to those genes that had previously been shown to contribute to AML (or other malignancies) by their down regulation. *RB1CC1 *is a tumour suppressor and transcriptional promoter of key cell cycle regulator retinoblastoma-1 [53] that has been found to be inactivated in breast cancer by the intriguing means of truncational mutation [54]. Similarly, *TFAP2C *transcriptionally activates p21 expression, retards breast cancer cell growth, and decreases clonogenic survival [55]. *ID4 *is a putative tumour suppressor gene that is down-regulated by hypermethylation in numerous cancers including ALL [56] and MDS [57]. *KLF4 *is not only a critical regulator of monocytic differentiation [58], but has been shown to be pathologically inactivated in medulloblastoma [59], and recently to interact with miR-10b in transition to oesophageal cancer [22]. *NR4A3 *has previously been demonstrated to be a critical tumor suppressor of myeloid leukemogenesis [60] and possesses two 3'UTR seed matches to miR-10a, which may increases capacity for translational repression [61]. *ARNT *was examined, since down-regulation of the *AHR*/*ARNT *pathway by epigenetic means in acute lymphoblastic leukaemia (ALL) [62] may be extrapolatable to myeloid disease. *GTF2H1 *is a general transcription factor involved in nucleotide excision repair, contributing to the maintenance of genomic stability [63]. *MAPRE1 *is involved in microtubular physiology and maintenance of chromosomal stability [64], processes often disturbed in AML. We have demonstrated that these genes were shown to be the suppressible targets of miR-10a by report assays and warrant further investigation. The mechanism of miR-10a over-expression is unknown. It could be consequent upon copy number amplification at the 17q21.32 locus where miR-10a resides, although this was not seen by high density SNP array (at 1 megabase resolution) in a cohort of AML patients which included 14 cases of *NPM1*-AML [65]. miR-10a expression is influenced by the methylation status of an upstream promoter [46], therefore analysis of the methylation status and the broader epigenetic state of the miR-10a locus in *NPM1^mut^*-AML would be helpful.

## Conclusions

We have shown that miR-10a is markedly over-expressed in *NPM1^mut^*-AML, and provided new evidence that miR-10a is the dominantly expressed miR-10 family member in this class of AML. *NPM1^mut ^*OCI-AML3 cell line shares high miR-10a expression with its primary AML derivative. Knockdown of miR-10a in these cells results in altered growth and cellular survival. In view of these findings as well as those in other malignancies, further evaluation of the role of miR-10a in *NPM1*^mut ^AML is warranted.

## Methods

### Patient samples

The diagnostic bone marrow samples of 28 NK-AML cases with pre-Ficoll marrow blast counts of at least 50% were obtained after informed consent from the tissue banks of our institute and the Australian Leukaemia and Lymphoma Group Tissue Bank (demographic data shown in Additional file 4: Table S2). Eight normal bone marrow samples were randomly obtained from our institute's tissue bank for comparison. To determine *NPM1 *mutational status, the insertional hotspot was RT-PCR amplified from complementary DNA using previously described primers [66] and directly sequenced on the 3100XL from ABI 3100XL Genetic Analyser using the reverse primer. 19/28 (68%) samples harboured *NPM1^mut^*.

### microRNA microarray and qRT-PCR

RNA was extracted from post-ficoll bone marron mononuclear cells, and microRNA microarray (miRCURY LNA microRNA probe set, Cat # 208010V8.1, Exiqon, Vedbaek, Denmark) performed as previously described in an overlapping cohort of patients [67]. The microarray was performed at the Adelaide Microarray Facility. In brief, 5 μg of total fluorescent labelled RNA was analysed in duplicate by two-sample (dual colour) competitive hybridization with dye swap to control for labelling efficiency. Slides were scanned at 10 μm resolution with a Genepix 4000B Scanner (Molecular Devices, Sunnyvale, CA). For the reference channel the total RNA from normal patient samples were pooled in equal quantities.

For the bioinformatic microarray analysis, data were processed as described [15] and [68]. We obtained the fold change (FC) of microRNAs differentially expressed (DE) between *NPM1^mu^*^t ^and *NPM1^wt^*-AML samples. For this analysis we tested the null hypothesis that the difference in log-2 fold change for *NPM1^mu^*^t ^and *NPM1^wt ^*AML samples was 0 and the p-values were adjusted for multiple comparisons. Hierarchical cluster analysis was then performed using the probe sets identified to be significantly DE and a heat map was generated.

To confirm miR-10a expression in primary samples and comparison of expression in cell lines, 1 μg of total RNA was used for cDNA synthesis reaction using the NCode microRNA first strand synthesis and qRT-PCR kit (Invitrogen, USA). Quantitative real time polymerase chain reaction (qRT-PCR) was performed using the Platinum Sybr Green Taq (Invitrogen, USA) and the Rotorgene RG-3000 thermocycler (Qiagen, Hilden, Germany). RNU6b was used as the housekeeping microRNA. The specific qRT-PCR primers were synthesised to order by Integrated DNA Technologies (USA) as follows: miR-10a (5-taccctgtagatccgaatttgtg-3) and RNU6b (5-cgcttcggcagcacatatac-3). For functional cell line work, microRNA expression was quantified by the TaqMan miRNA assay (Applied Biosystems) as per manufacturer's instructions, on the Rotorgene RG-3000 thermocycler. All qPCR reactions were performed in duplicate and RNU6b was also used as the housekeeping microRNA.

### miR-10a antisense repression and functional assays in OCI-AML3 cells

The OCI-AML3 cell line is the only available AML cell line bearing the *NPM1 *gene mutation (type A TCTG duplication) and shares numerous phenotypic features with primary *NPM1^mut^*-AML samples (14). OCI-AML3 cells purchased from the DSMZ cell-line bank (Braunschweig, Germany) were cultured in α-MEM solution with 10% fetal bovine serum (GIBCO) once their *NPM1 *mutational status was confirmed. Cells were transfected with 50 nM anti-miR10a miRCURY LNA™ microRNA knockdown probes or negative control (Exiqon, Denmark) using Lipofectamine 2000 and Opti-MEM (both from Invitrogen, USA) in antibiotic-free media. Cells were maintained in a humidified incubator at 37°C in 5% CO2. miR-10a knockdown was confirmed by TaqMan qRT-PCR (Applied Biosystems) (Additional file 3: Figure S2).

Cell growth and viability was analysed at 24 and 48 h using the Promega CellTiter Aqueous One Solution Cell Proliferation Assay (Promega) according to the manufacturer's instructions, using Multiskan plate reader (Thermo Labsystems). For this assay cells were plated in triplicate for each transfection condition and time point. The MTS absorbance reading at each time point was normalised to a non-transfected lipofectamine-only control.

Cell cycle analysis was performed by exposing cells to 1 mL 1% TX-100, 50 μL 50 mg/mL propidium iodide (PI) and 50 μL of 10 mg/mL RNAase A at 37°C for 30 min and analysed by the LSRII flow cytometer (BD biosciences).

Apoptotic fractions of LNA-transfected OCI-AML3 cells were analysed by Annexin V/PI staining using the Annexin V-FITC Apoptosis Detection Kit I (BD Pharminogen) according to the manufacturer's instructions. Caspase-3 activation was measured using the plate-based Caspase-Glo^® ^3/7 Assay (Promega). Intracellular activated caspase-3 was also directly measured by staining permeabilised cells (FACSperm, BD Biosciences) with PE Rabbit Anti-Active Caspase-3 (BD Pharmingen). Analysis was performed on FlowJo Software on at least 10,000 events acquired using a LSRII flow cytometer (BD Biosciences).

Clonogenicity of OCI-AML3 cells treated with anti-miR10a LNA or negative control was assayed by seeding 400 OCI-AML3 cells in 1 mL 1% MethoCell MC (in RPMI with 10% FBS per 35 mm plate). Colony number and size were scored microscopically (colony defined as > 50 cells) using standard criteria after 14 days at 37°C in 5% CO_2_.

### miR-10a target prediction and validation

miRanda (union of http://microRNA.org and miRBase), PicTar and TargetScan algorithms were utilised for the *in silico *prediction of miR-10a mRNA targets. The DAVID Functional Annotation Tool (http://david.abcc.ncifcrf.gov/summary.jsp) was used to classify the genes into ontologically-related terms and then examine for potentially enriched terms [69]. To identify genes of functional relevance in AML, several resources were utilised. To capture potential oncogenes or tumour suppressor genes, the Sanger Cancer Gene Census [70] was sourced. Next, the following Kyoto Encyclopaedia of Genes and Genomes (KEGG) Pathways [71] were considered: AML (hsa05221), apoptosis (hsa04210) and cell cycle (hsa04110). To capture genes related to myeloid differentiation, the following terms annotated by the Gene Ontology Consortium (GOC) [72] were considered: haematopoiesis (GO:0030097), myeloid cell differentiation (GO:0030099), myeloid leukocyte differentiation (GO:0002573) and several terms related to the regulation of these pathways (GO:0045637, GO:0045638, GO:0045639, GO:0002761, GO:0002762 and GO:0002763). Finally advantage was taken of published *NPM1^mut^*-AML gene expression profiling data [15-17] by compiling a list of genes down-regulated in *NPM1^mut^*-AML on the microarrays.

Bioinformatically predicted miR-10a binding sites were analysed by luciferase reporter assay as described in detail previously [67]. To confirm the specificity of the 3'UTR binding site, at least 3 bases of the relevant seed region in the pMIR.3UTR constructs were mutated by site-directed mutagenesis (Stratagene) or by a PCR-based technique and the assay was repeated using the mutated construct.

## Abbreviations

AML: Acute myeloid leukaemia; NPM1: Nucleophosmin-1; NK: Normal karyotype; LNA: Locked nucleic acid.

## Competing interests

The authors declare that they have no competing interests.

## Authors' contributions

AB carried out qRT-PCR, apoptosis, cell cycle and cell growth assays, microRNA target prediction and validation by luciferace assay, and wrote the manuscript. CAP carried out the clonogenic, BrdU, differentiation assays and contributed to the apoptosis assays and wrote the manuscript. VY performed the bioinformatic microarray analysis. YWY contributed to statistical analysis. ML carried out the microarrays, participated in the design of the study and wrote the manuscript. DM conceived of the study, participated in its design, supervised the experiments and helped to write the manuscript. All authors read and approved the final manuscript.

## Supplementary Material

Additional file 1 **Table S1. List of significantly (p < .05) differentially expressed probes on microarray comparison of NK-AML samples versus normal bone marrow**. Only those microRNAs with a FC of ≥ 2 in either direction were included in this table. There were 26 overexpressed probes and 11 under expressed probes in NK-AML versus normal BM. The microRNAs depicted in bold represent proprietary miRPlusTM probe whose sequences have subsequently been annotated on miRBase. Those probes denoted as miRPlusTM have not been further annotated by the release of miRBase release 15. Click here for file

Additional file 2 **Figure S1. Confirmation of miR-10a knockdown by anti-10a LNA**. **A**. miR-10a expression 24 h after transfection with Exiqon (LNA) anti-microRNA ASO was assayed by TaqMan microRNA qRT-PCR. RNU6b was used as the reference gene. MiR-10a expression was normalised to that of cells transfected with 100 nM of the non-targeting control specific to each chemistry, with comparison made to untransfected cells The graph depicts the mean miR-10a relative expression of individual experiments for Ambion Anti-miRs (n = 4) and Exiqon LNAs (n = 3), +/-SEM (of fold change values). **B**. Hela cells were pre-treated with either pcDNA.10a (miR-10a overexpressing plasmid), 30 nM of Pre-miR-10a or were not pre-treated. After 6 h, transfection media was removed and cells washed with PBS. Cells were then transfected with pMIR.HOXA1/pRLCMV and 50 nM of either anti-10a LNA or LNA Control A. After 24 h, Dual Luciferase Assay (Promega) was performed in triplicate. The whole experiment was repeated 5 times. The graph depicts the mean luciferase values for anti-10a LNA treated cells compared to the LNA Control A treated cells (+/-SEM), with the control values corrected to 100. The statistical analysis consists of Student's t-test (paired). NS: not significant; * 0.01 < p < 0.05; ** 0.001 < p < 0.01. Click here for file

Additional file 3 **Figure S2. Monocytic differentiation of OCI-AML3 cells is not affected by miR-10a knockdown**. 1,25-dihydroxyvitaminD3 (VitD3) was used to induce monocytic differentiation of OCI-AML3 cells over a 96 h period. **A**. Morphological analysis (Wright Stain) of cytospin samples of OCI-AML3 cells treated with VitD3 demonstrated no observable differences between SCRAM control transfected and anti-miR10a LNA transfected groups. **B**. Phenotype analysis of CD14 **C**. CD15 and **D**. CD11b expression by flow cytometry did not detect a statistically significant difference between SCRAM control or anti-miR10a LNA groups at 48 h or 96 h post, regardless if cells were treated with VitD3 (+) or did not receive treatment (-). N = 3. Click here for file

Additional file 4 **Table S2**. Patient demographics and AML blast characteristics. Click here for file
