# Supplementary material for: miR-10a is aberrantly overexpressed in Nucleophosmin1 mutated acute myeloid leukaemia and its suppression induces cell death
Source: Mol Cancer. 2012 Feb 20;11:8. doi: 10.1186/1476-4598-11-8 (PMC3306826; doi:10.1186/1476-4598-11-8)
Supplement: Additional file 1 — Table S1. List of significantly (p < .05) differentially expressed probes on microarray comparison of NK-AML samples versus normal bone marrow. Only those microRNAs with a FC of ≥ 2 in either direction were included in this table. There were 26 overexpressed probes and 11 under expressed probes in NK-AML versus normal BM. The microRNAs depicted in bold represent proprietary miRPlusTM probe whose sequences have subsequently been annotated on miRBase. Those probes denoted as miRPlusTM have not been further annotated by the release of miRBase release 15. [file 1476-4598-11-8-S1.PDF]

Supplementary Table 1: Patient demographics and AML blast characteristics.

28 AML samples were analysed by microRNA microarray. 12 samples were from the St Vincent's Hospital (SVH) tissue bank and 16 were from the ALLG tissue bank. The FAB subtype was assigned to all patient samples as indicated. Peripheral blood (PB) leukocyte total and blast cell counts at initial presentation are detailed. Induction therapy was either with a standard (200mg/m^2 for 7 days) or high (>1g/m^2 for 8 doses) or was not offered (nil). First complete remission (CR1) was defined as being attained by those patients with <5% blasts on bone marrow biopsy at haematopoietic recovery from induction chemotherapy

Sample Number	Age of Patient	Sex of Patient	Morphology (FAB)	% Marrow Blasts	PB Leukocyte Count	PB Blast Count	Induction Therapy	CR1 attained	NPM1 status	FLT3 Status
1	76	F	M5b	81	75	9	standard	yes	mut	wt
2	81	M	M4a	82	5	0	nil	no	wt	wt
3	23	F	M5	73	6	0	high	yes	mut	mut
4	67	M	M1	89	44	40	standard	yes	wt	wt
5	76	F	M1	61	9	8	nil	no	mut	wt
6	75	F	M2	78	13	2	nil	no	mut	mut
7	57	F	M1	85	83	74	standard	yes	mut	mut
8	70	M	M1	80	5	3	standard	yes	wt	wt
9	41	M	M2	90	158	155	standard	yes	mut	wt
10	33	M	M5b	90	28	7	high	yes	mut	wt
11	49	M	M5	84	27	2	high	yes	mut	wt

			b							
12	56	M	M5<br>b	77	77	7	high	yes	mut	wt
13	59	M	M4	63	63	46	high	yes	mut	mut
14	59	F	M2	66	8	1	high	yes	mut	wt
15	40	M	M4	80	104	14	high	yes	mut	wt
16	46	M	M1	89	4	2	high	yes	mut	wt
17	45	F	M2	67	5	3	high	yes	wt	wt
18	56	M	M1	90	98	88	high	yes	wt	wt
19	48	F	M5	72	6	1	high	yes	mut	wt
20	41	F	M5<br>b	67	73	19	high	yes	mut	wt
21	48	M	M1	93	104	104	high	yes	wt	wt
22	46	F	M4	52	162	70	high	yes	mut	mut
23	48	M	M2	61	3	1	high	yes	wt	wt
24	44	F	M4	64	20	4	high	yes	mut	mut
25	51	M	M4	74	75	33	high	yes	mut	mut
26	46	M	M2	62	21	15	high	yes	mut	mut
27	39	F	M2	55	5	2	high	no	wt	mut
28	35	M	M2	59	1	0	high	yes	wt	wt
