# Supplementary material for: miR-10a is aberrantly overexpressed in Nucleophosmin1 mutated acute myeloid leukaemia and its suppression induces cell death
Source: Mol Cancer. 2012 Feb 20;11:8. doi: 10.1186/1476-4598-11-8 (PMC3306826; doi:10.1186/1476-4598-11-8)

Supplementary Figure 1: Confirmation of miR-10a knockdown by anti-10a LNA. **A**. miR-10a expression 24 hours after transfection with Exiqon (LNA) anti-microRNA ASO was assayed by TaqMan microRNA qRT-PCR. RNU6b was used as the reference gene. MiR-10a expression was normalised to that of cells transfected with 100nM of the non-targeting control specific to each chemistry, with comparison made to untransfected cells The graph depicts the mean miR-10a relative expression of individual experiments for Ambion Anti-miRs (n=4) and Exiqon LNAs (n=3), +/-SEM (of fold change values). **B**. Hela cells were pre-treated with either pcDNA.10a (miR-10a overexpressing plasmid), 30nM of Pre-miR-10a or were not pre-treated. After 6 hours, transfection media was removed and cells washed with PBS. Cells were then transfected with pMIR.HOXA1/pRL-CMV and 50nM of either anti-10a LNA or LNA Control A. After 24 hours, Dual Luciferase Assay (Promega) was performed in triplicate. The whole experiment was repeated 5 times. The graph depicts the mean luciferase values for anti-10a LNA treated cells compared to the LNA Control A treated cells (+/-SEM), with the control values corrected to 100. The statistical analysis consists of Student's t-test (paired). NS: not significant; * 0.01<p<0.05; ** 0.001<p<0.01.

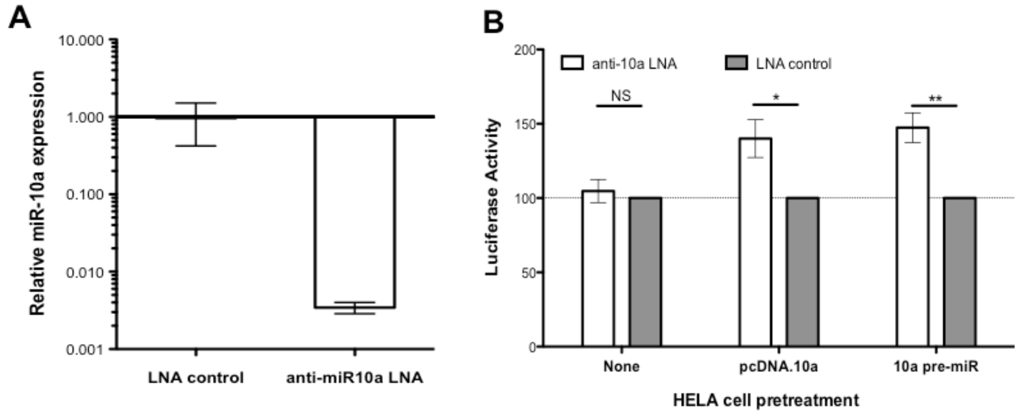

Supplement: Additional file 2 — Figure S1. Confirmation of miR-10a knockdown by anti-10a LNA. A. miR-10a expression 24 h after transfection with Exiqon (LNA) anti-microRNA ASO was assayed by TaqMan microRNA qRT-PCR. RNU6b was used as the reference gene. MiR-10a expression was normalised to that of cells transfected with 100 nM of the non-targeting control specific to each chemistry, with comparison made to untransfected cells The graph depicts the mean miR-10a relative expression of individual experiments for Ambion Anti-miRs (n = 4) and Exiqon LNAs (n = 3), +/-SEM (of fold change values). B. Hela cells were pre-treated with either pcDNA.10a (miR-10a overexpressing plasmid), 30 nM of Pre-miR-10a or were not pre-treated. After 6 h, transfection media was removed and cells washed with PBS. Cells were then transfected with pMIR.HOXA1/pRLCMV and 50 nM of either anti-10a LNA or LNA Control A. After 24 h, Dual Luciferase Assay (Promega) was performed in triplicate. The whole experiment was repeated 5 times. The graph depicts the mean luciferase values for anti-10a LNA treated cells compared to the LNA Control A treated cells (+/-SEM), with the control values corrected to 100. The statistical analysis consists of Student's t-test (paired). NS: not significant; * 0.01 < p < 0.05; ** 0.001 < p < 0.01. [file 1476-4598-11-8-S2.PDF]
