# Supplementary material for: miR-10a is aberrantly overexpressed in Nucleophosmin1 mutated acute myeloid leukaemia and its suppression induces cell death
Source: Mol Cancer. 2012 Feb 20;11:8. doi: 10.1186/1476-4598-11-8 (PMC3306826; doi:10.1186/1476-4598-11-8)

Supplementary Figure 2: Monocytic differentiation of OCI-AML3 cells is not affected by miR-10a knockdown. 1,25-dihydroxyvitaminD3 (VitD3) was used to induce monocytic differentiation of OCI-AML3 cells over a 96 hour period. **A**. Morphological analysis (Wright Stain) of cytospin samples of OCI-AML3 cells treated with VitD3 demonstrated no observable differences between SCRAM control transfected and anti-miR10a LNA transfected groups. **B**. Phenotype analysis of CD14 **C**. CD15 and **D**. CD11b expression by flow cytometry did not detect a statistically significant difference between SCRAM control or anti-miR10a LNA groups at 48hr or 96hrs post, regardless if cells were treated with VitD3 (+) or did not receive treatment (-). N=3.

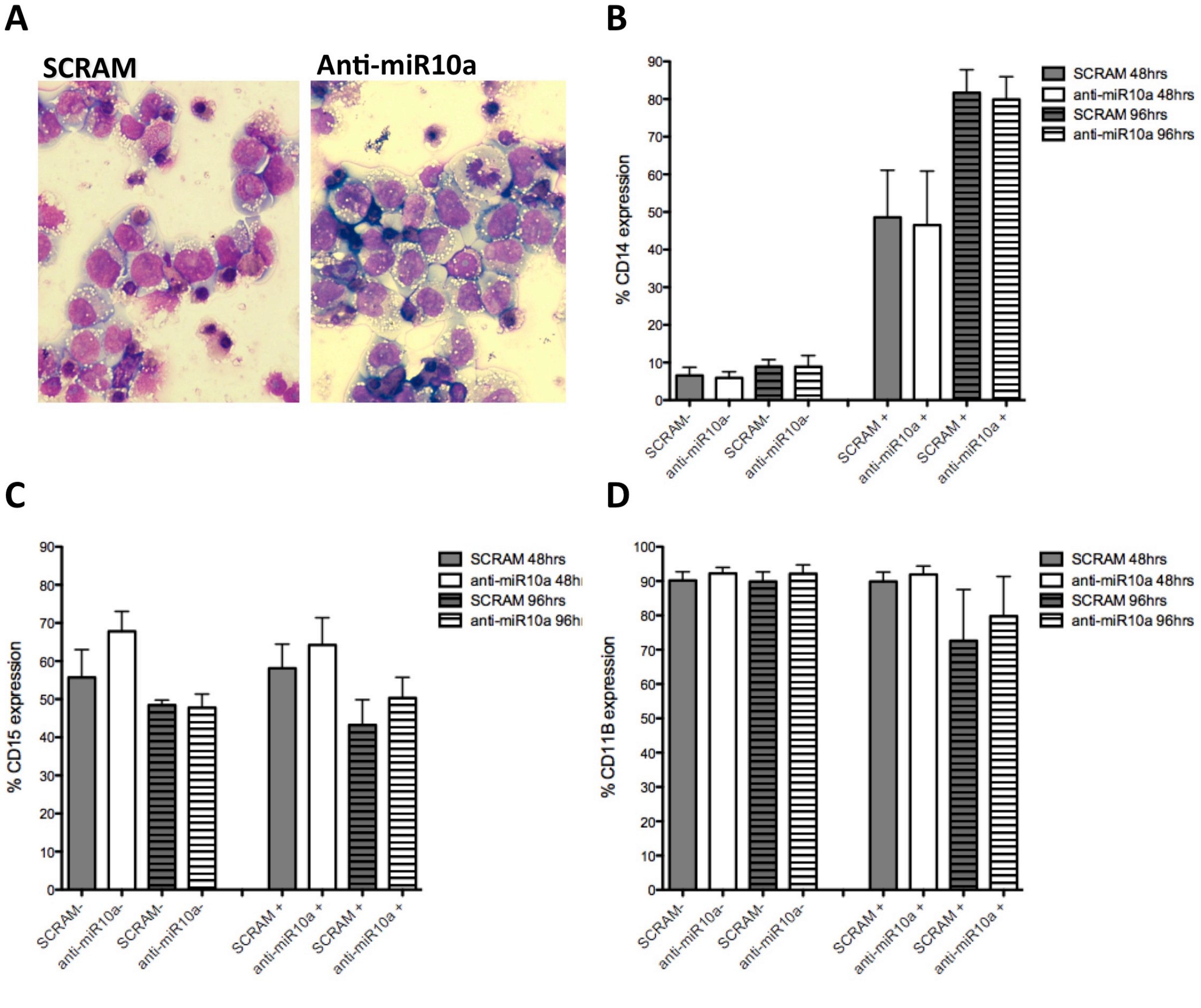

Supplement: Additional file 3 — Figure S2. Monocytic differentiation of OCI-AML3 cells is not affected by miR-10a knockdown. 1,25-dihydroxyvitaminD3 (VitD3) was used to induce monocytic differentiation of OCI-AML3 cells over a 96 h period. A. Morphological analysis (Wright Stain) of cytospin samples of OCI-AML3 cells treated with VitD3 demonstrated no observable differences between SCRAM control transfected and anti-miR10a LNA transfected groups. B. Phenotype analysis of CD14 C. CD15 and D. CD11b expression by flow cytometry did not detect a statistically significant difference between SCRAM control or anti-miR10a LNA groups at 48 h or 96 h post, regardless if cells were treated with VitD3 (+) or did not receive treatment (-). N = 3. [file 1476-4598-11-8-S3.PDF]
