# Supplementary material for: miR-10a is aberrantly overexpressed in Nucleophosmin1 mutated acute myeloid leukaemia and its suppression induces cell death
Source: Mol Cancer. 2012 Feb 20;11:8. doi: 10.1186/1476-4598-11-8 (PMC3306826; doi:10.1186/1476-4598-11-8)
Supplement: Additional file 4 — Table S2. Patient demographics and AML blast characteristics. [file 1476-4598-11-8-S4.PDF]

Supplementary Table 2: List of significantly (p<.05) differentially expressed probes on microarray comparison of NK-AML samples versus normal bone marrow. Only those microRNAs with a FC of ≥2 in either direction were included in this table. There were 26 overexpressed probes and 11 under expressed probes in NK-AML versus normal BM. The microRNAs depicted in bold represent proprietary miRPlus^{TM} probe whose sequences have subsequently been annotated on miRBase. Those probes denoted as miRPlus^{TM} have not been further annotated by the release of miRBase release 15.

**Upregulated miRNAs**		**Downregulated miRNAs**	
**miR-probe**	**FC**	**miR-probe**	**FC**
miR-10a	19.6	miR-451	-5.7
let-7b	6.0	miR-22	-3.8
miR-20a	4.8	miR-plus (17856)	-3.0
let-7c	4.7	miR-plus (17952)	-2.8
miR-222	4.6	miR-483	-2.5
miR-221	4.4	**miR-125-3p**	-2.5
miR-17-5p	4.3	miR-21	-2.3
miR-196b	4.3	miR-150	-2.2
mir-20a	3.8	**miR-744**	-2.1
let-7a	3.8	miR-plus (17881)	-2.1
miR-181a	3.7	miR-342	-2.0
**miR-886-5p**	3.5		
miR-363*	3.5		
miR-181b	3.3		
miR-155	2.8		
miR-plus (17848)	2.7		
miR-34a	2.7		
miR-20b	2.7		
**miR-223***	2.6		
miR-19b	2.5		
miR-181a	2.5		
**miR-221***	2.4		
miR-191	2.3		
miR-17-3p	2.1		
miR-345	2.1		
miR-plus (17818)	2.0		
